# A Doppler Transient Model Based on the Laplace Wavelet and Spectrum Correlation Assessment for Locomotive Bearing Fault Diagnosis

**DOI:** 10.3390/s131115726

**Published:** 2013-11-18

**Authors:** Changqing Shen, Fang Liu, Dong Wang, Ao Zhang, Fanrang Kong, Peter W. Tse

**Affiliations:** 1 Department of Precision Machinery and Precision Instrumentation, University of Science and Technology of China, Hefei 230026, China; E-Mails: shenchangqing@mail.ustc.edu.cn (C.S.); liufang1@mail.ustc.edu.cn (F.L.); zhangao@mail.ustc.edu.cn (A.Z.); 2 Department of Systems Engineering and Engineering Management, City University of Hong Kong, Hong Kong; E-Mails: dongwang4-c@my.cityu.edu.hk (D.W.); meptse@cityu.edu.hk (P.W.T.)

**Keywords:** Doppler transient model, locomotive bearings, spectrum correlation assessment, Laplace wavelet, fault diagnosis

## Abstract

The condition of locomotive bearings, which are essential components in trains, is crucial to train safety. The Doppler effect significantly distorts acoustic signals during high movement speeds, substantially increasing the difficulty of monitoring locomotive bearings online. In this study, a new Doppler transient model based on the acoustic theory and the Laplace wavelet is presented for the identification of fault-related impact intervals embedded in acoustic signals. An envelope spectrum correlation assessment is conducted between the transient model and the real fault signal in the frequency domain to optimize the model parameters. The proposed method can identify the parameters used for simulated transients (periods in simulated transients) from acoustic signals. Thus, localized bearing faults can be detected successfully based on identified parameters, particularly period intervals. The performance of the proposed method is tested on a simulated signal suffering from the Doppler effect. Besides, the proposed method is used to analyze real acoustic signals of locomotive bearings with inner race and outer race faults, respectively. The results confirm that the periods between the transients, which represent locomotive bearing fault characteristics, can be detected successfully.

## 1. Introduction

Economic and social development in most countries has increased considerably the requirement for transportation capability. Railway transportation has played an important role in this development due to its strong transportation capability and high speeds. The continuous operation of trains is crucial in ensuring fluid and efficient traffic circulation. However, failure of train components can result in unexpected breakdowns, which can lead to serious traffic accidents. Hence, both the economy and human safety are at risk if trains have faulty components. Locomotive bearings support the entire weight of a train and they rotate at a high speed when the train is running. The health of these bearings is crucial for the continuous and safe operation of the train. Therefore, the development of an effective technique for monitoring locomotive bearings is profoundly significant [1].

A bearing usually consists of an inner race, an outer race, a cage, and a few rollers. Once one of these components suffers from a local defect, approximately periodic impacts will be generated when the defective surface comes into contact with the rollers [2]. These transient interaction components therefore contain important information about the health status of the bearing. Extracting these components is the most important task in bearing fault diagnosis based on signal processing [3].

The wayside acoustic defective bearing detector (ADBD) system [4] was developed in the 1980s to identify bearing defects before the bearings are overheated. All the devices in this system are set on the wayside, which makes the system more economical and feasible compared to an on-board monitoring system [5]. Through the ADBD system, the health status of locomotive bearings can be detected in passing vehicles. However, when the sound source is moving relative on the microphone, the Doppler effect will occur in the recorded signals. The signals obtained by the ADBD system will suffer from high frequency shift, frequency band expansion, and amplitude modulation [6], causing a significant decline in the performance of the system, particularly when the vehicles pass at high speeds.

Various methods have been developed for bearing fault diagnosis when no relative movement is observed between the bearing and the data acquisition system. Time-frequency analysis, which can extract information from both the time and the frequency domains, was developed for non-stationary signals. Several representative time-frequency distributions [7,8], such as the Wigner-Ville and the Choi-Williams distributions, have proven their potential in bearing fault signal processing [9]. Wavelet transforms were developed to decompose a temporal raw signal into different scales with varying frequency bandwidths [10,11]. Thus, wavelet transforms can be used to enhance bearing fault-related information for further processing [12,13]. The ensemble empirical mode decomposition (EEMD) is an adaptive decomposition method that can decompose nonlinear and non-stationary signals into a set of intrinsic mode functions (IMFs) according to its own natural oscillatory modes [14] and has been widely applied in diagnosing bearing faults [15,16].

Matching pursuit is an adaptive approach that selects optimal atoms to approximate a signal through iterations. It is effective for analyzing bearing fault transient signals [17]. Freudinger *et al.* [18] introduced a correlation filtering approach that uses vector inner products between a time history and a set of Laplace wavelets as a measure of the correlation between the data and a range of modal dynamics characterized by the wavelets. The Laplace wavelet parameters, through which the local maxima are derived, are regarded as the closest to the observed the model parameters of the system. Based on these fundamentals, Wang *et al.* [19,20] proposed a method that incorporates a transient model and parameter identification based on wavelets and correlation filtering to achieve bearing fault feature detection. However, high frequency shifts, frequency band expansions, and amplitude modulations occur in the wayside ADBD system due to the Doppler effect. The discussed techniques cannot be applied directly to this problem.

In this paper, a novel technique that combines a Doppler transient model and parameter identification based on the Laplace wavelet and a spectrum correlation assessment is proposed for real locomotive bearing fault detection. The Doppler transient model is constructed by considering the effect of Doppler distortion. Model parameters, including the transient periods, are identified by a correlation assessment between the envelope spectrum of the transient model and the real bearing fault signal. The results obtained through both simulations and real case studies demonstrate the remarkable performance of the technique in identifying locomotive bearing fault types.

The rest of this paper is organized as follows: Section 2 briefly describes the fundamental theory that underlies the Laplace wavelet and the correlation assessment. The proposed method is presented in Section 3, followed by the simulation analysis and the real case study in Section 4. Conclusions are presented in Section 5.

## 2. Theoretical Background

### 2.1. Transient Model Based on the Laplace Wavelet

During defective bearing movement, periodic impacts occur in the obtained signals. These transient components can be matched by using elements in a model dictionary. Five representative transient models are usually used to simulate transient components caused by bearing faults, the Morlet wavelet, Harmonic wavelet, Laplace wavelet, single-side Morlet wavelet, and single-side Harmonic wavelet. The Laplace wavelet is a single-sided damped exponential function formulated as the impulse response of a single mode system. It is similar to the waveform feature commonly encountered in bearing fault signal detection tasks [19]. A transient model based on the Laplace wavelet is therefore used for further analysis. The formula of the real part of the Laplace wavelet is given as:

(1) $$\psi_{\text{Laplace}}(f,\zeta ,\tau ,t)=\{\begin{array}{c} \begin{array}{cc} e^{-\zeta /\sqrt{1-\zeta^{2}}2\pi f\times (t-\tau )} & \cos(2\pi f\times (t-\tau )),\tau \leq t\leq \tau +W\\ 0 & \text{else} \end{array} \end{array}$$

where W is the temporal range, *f* is the discrete frequency, ζ is the discrete damping coefficient, and *τ* is the discrete delay time. These parameters belongs to subset *F*, *Z*, and *T_d_* as shown below:

(2) $$\begin{array}{c} F\subset R^{+}\\ Z\subset (R^{+}\cap [0,1))\\ T_{d}\subset R^{+} \end{array}$$

A periodic multi-transient model based on the Laplace wavelet is constructed to simulate the waveform characteristics by introducing parameter *T*:

(3) $$\chi \left(t\right)=\sum_{m}\psi_{\text{Laplace}}(f,\zeta ,\tau ,t-mT)$$

**Figure 1.**
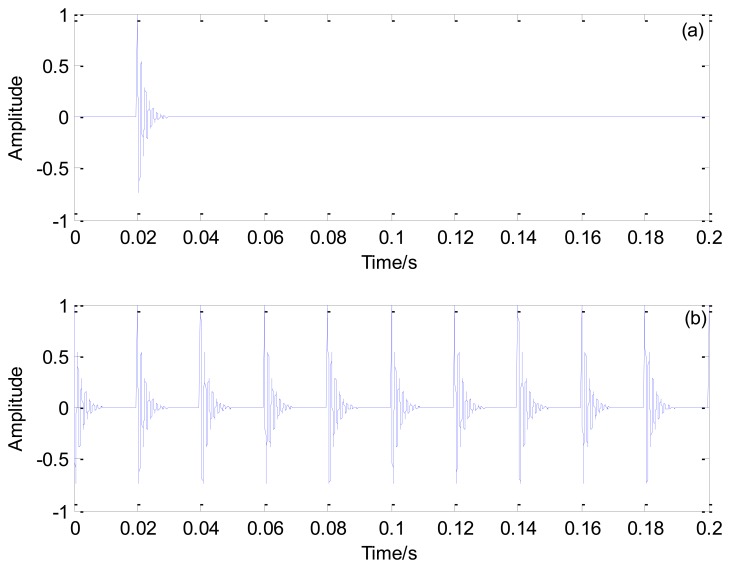
(**a**) Single transient and (**b**) periodic Laplace wavelet transient model.

Figure 1 illustrates the single and periodic Laplace wavelet transient models, respectively.

### 2.2. Correlation Analysis

In mathematics, the inner product serves as a powerful tool for evaluating the similarity of two time series. Suppose that two time series *x(n)* and *y(n)* have the same length *N*. Then, the inner product operation 〈 · 〉 for the two finite length signals can be represented as [21]:

(4) $$\langle x(n),y(n)\rangle =\sum_{i=1}^{N}x(i)\times y(i)$$

The correlation coefficient *ρ_x_*_(_*_n_*_),_*_φ_γ__*_(_*_n_*_)_, based on the inner product, can be used to assess the degree of correlation between the two time series. Its formula is given by:

(5) $$\rho_{x(n),\psi_{\gamma }(n)}=\begin{array}{cc} \frac{\langle x(n),\psi_{\gamma }(n)\rangle }{\sqrt{\langle x(n),x(n)\rangle }\sqrt{\langle \psi_{\gamma }(n),\psi_{\gamma }(n)\rangle }} & =\frac{\overset{N}{\underset{i=1}{\text{∑}}}x(i)\times \psi_{\gamma }(i)}{\sqrt{\overset{N}{\underset{i=1}{\text{∑}}}x(i)\times x(i)}\sqrt{\overset{N}{\underset{i=1}{\text{∑}}}\psi_{\gamma }(i)\times \psi_{\gamma }(i)}} \end{array}$$

In terms of the Cauchy-Schwarz inequality, the correlation coefficient is constrained to:

(6) $$-1\leq \rho_{x(n),\psi_{\gamma }(n)}\leq 1$$

When the correlation coefficient is closer to 0, the linear dependence relationship between the two signals is weaker.

## 3. Proposed Doppler Transient Model Based on Laplace Wavelet and Spectrum Correlation Assessment

**Figure 2.**
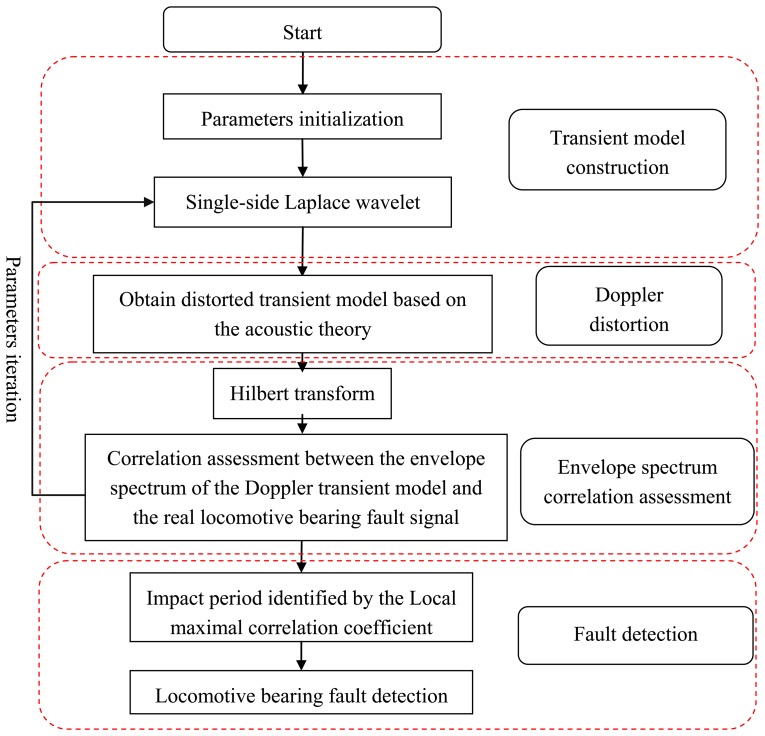
Flowchart of the proposed locomotive bearing fault diagnosis.

The conventional bearing fault detection methods have been developed for situations with no relative movement between the signal acquisition system and the defective bearing, and thus the acquired signal is not affected by the Doppler effect, however, locomotive bearing signals suffer from high frequency shifts, frequency band expansions, and amplitude modulations due to the Doppler effect. The fault-related impact intervals are not identical in this situation. Hence, the conventional detection methods are not applicable in the diagnosis of real locomotive bearing faults. In this study, a Doppler transient model based on the Laplace wavelet and a spectrum correlation assessment is proposed to address the inability of traditional methods to handle Doppler-distorted acoustic signals in real locomotive bearing fault detection. The correlation coefficient in the frequency domain does not need to consider the transient model's time delay in this method, reducing the computation time required for parameter identification and thus improving the computational efficiency. A flowchart of the proposed scheme is shown in Figure 2.

The proposed method follows the steps of transient model construction, Doppler distortion, parameter identification through the assessment of the envelope spectrum correlation, and bearing fault type identification through the recognized impact periods. Each step is discussed in detail in the following subsections.

### 3.1. Doppler Distortion of the Transient Model Based on the Laplace Wavelet

**Figure 3.**
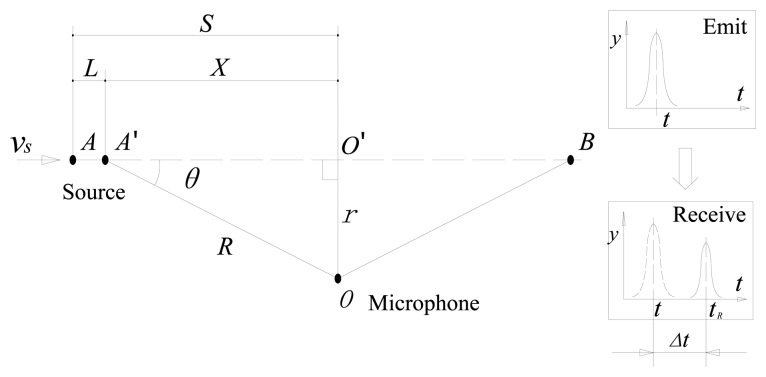
Depiction of a single sound source moving in a straight line.

The Doppler effect was first proposed in 1842 by Austrian physicist Christian Doppler [22]. As shown in Figure 3, *S* is the distance between the initial position and the position when the sound source passes by the microphone. *L* is the current displacement. *X* is the distance between the current position and the position when the sound source passes by the microphone. *R* is the distance between the source point and the microphone. A time delay exists due to the distance between the sound source and the microphone. When the sound source has a movement speed *V_s_* relative to the receiver, the wave frequency changes for the receiver. The observed frequency is higher than the emission frequency during the source's approach, is identical at the instant when the source passes by, and is lower during the source's departure.

The Doppler effect makes traditional techniques unsuitable for processing locomotive bearing signals. To address this problem, the Doppler transient model is constructed for further analysis. The Doppler effect is embedded manually into the conventional transient model so that the constructed model is under the same distortion environment as the real locomotive bearing signal. According to acoustic theory, the following formula and procedures can be proposed:

(1) Calculating the emission and reception time instants: The reception time instants {*t_R_*} = {*t*_0_, *t*_0_ + 1/*f_s_*, *t*_0_ + 2/*f_s_*, *t*_0_ + *(N*−*1)*/*f_s_*} are assumed, where *f_s_* is the sampling frequency, *N* is the data length, and *t_0_* is the initial time instant. As shown in Figure 3, the relationship between the emission and reception time instants can be represented as:
  (7) $$t_{R}=t_{e}+\Delta t=t_{e}+R/V_{sw}=t_{e}+\sqrt{r^{2}+{\left(S-L\right)}^{2}}/V_{sw}$$
  where *V_sw_* is the velocity of the sound waves in the medium, *t_e_* is the emission time instants, and *r* is the distance between the microphone and the line corresponding to the direction of the velocity of the sound source. *L* can be obtained by:
  (8) $$L(t)=\int_{0}^{t}V_{s}dt$$
(2) Interpolation: The periodic transient model *χ*(*t*) is interpolated in Equation (3) by using the emission time instants *t_e_*, which were calculated in Step 1 through a cubic spline. Let *χ_e_*(*t_e_*) represent the interpolated amplitude vector.
(3) Amplitude modulation: The amplitudes of the waveform are modulated during transmission from the moving sound source to the microphone. As introduced by Morse acoustic theory [23], it is assumed that the locomotive bearing moves with subsonic velocity (*M* = *V_s_*/*V_sw_* < 0.2), which indicates that the sound source is a monopole point source. Supposing that the medium has no viscosity, the received sound pressure can be expressed as:
  (9) $$P=\frac{q^{\prime }[t-(R/V_{sw})]}{4\pi R{\left(1-M\cos\theta \right)}^{2}}+\frac{q[t-(R/V_{sw})][\cos\theta -M]V_{s}}{4\pi R^{2}{\left(1-M\cos\theta \right)}^{3}}$$
  where *q* represents the total quality flow rate of the source point, *q′* is the derivative of *q*, *t* denotes the running time, θ represents the angle between the forward velocity of the sound source and the line from the sound source to the microphone, and *M* = *V_s_*/*V_sw_* is the Mach number of the source point's velocity.
  As shown in Equation (9), the received sound pressure comprises the near-field effect and the inverse relationship between the sound pressure and the distance between the source point and the microphone. When *M* < 0.2, the near-field effect can be neglected [24]. The received sound pressure is then given by:
  (10) $$P=\frac{q^{\prime }[t-(R/V_{sw})]}{4\pi R{\left(1-M\cos\theta \right)}^{2}}$$
  which can also be written as:
  (11) $$P=\frac{r}{R{\left(1-M\cos\theta \right)}^{2}}\cdot \frac{q^{\prime }[t-(R/V_{sw})]}{4\pi r}$$
  Where *r*/(*R*(*1* − *M*cos*θ*)^2^) is the amplitude modulation function and *q′*[*t*−(*R*/*V_sw_*)]/(4*πr*) is the received sound pressure when the microphone and the source point are both fixed. Therefore, the amplitude of the received waveform can be written as:
  (12) $$\chi_{R}=\chi_{e}\cdot \frac{r}{R{\left(1-M\cos\theta \right)}^{2}}$$

The Doppler effect is thus embedded in the constructed transient model to ensure that the Doppler transient model experiences the same distortion as the real locomotive bearing signal.

### 3.2. Envelope Spectrum Correlation Assessment

To simulate the characteristics of the received waveform in the fault signal of the locomotive bearing, the parameters of the constructed Doppler transient model must be adjusted to match the actual periodic impacts in the locomotive bearing signal. A suitable criterion must be established to optimize the parameters from the subsets, as shown in Equation (2). A new strategy to assess the envelope spectrum correlation is proposed as a quantitative measure to determine the optimal parameters. This strategy comprises three procedures:

(1) The Hilbert transforms of the periodic Doppler transient model and the real locomotive bearing fault signal are obtained [25]:
  (13) $$\begin{array}{c} H\left[\chi_{R}\left(t\right)\right]=\frac{1}{\pi }\int_{-\infty }^{\infty }\frac{\chi_{R}\left(t\right)}{t-\tau }d\tau \\ H\left[x_{A}\left(t\right)\right]=\frac{1}{\pi }\int_{-\infty }^{\infty }\frac{x_{A}\left(t\right)}{t-\tau }d\tau \end{array}$$
  where *x_A_(t)* is the real locomotive bearing fault signal, and the envelope signals are obtained by calculating the modulus of the analytic signals:
  (14) $$\begin{array}{c} E_{R}\left(t\right)=\left|A^{*}\left[\chi_{R}\left(t\right)\right]\right|=\left|\chi_{R}\left(t\right)+iH\left[\chi_{R}\left(t\right)\right]\right|\\ E_{A}\left(t\right)=\left|A^{*}\left[x_{A}\left(t\right)\right]\right|=\left|x_{A}\left(t\right)+iH\left[x_{A}\left(t\right)\right]\right| \end{array}$$
(2) Frequency spectrum analysis is performed by:
  (15) $$\begin{array}{c} ES_{R}\left(f\right)=\left|\int_{-\infty }^{\infty }E_{R}\left(t\right)e^{-i2\pi ft}dt\right|\\ ES_{A}\left(f\right)=\left|\int_{-\infty }^{\infty }E_{A}\left(t\right)e^{-i2\pi ft}dt\right| \end{array}$$
(3) The degree of correlation of the envelope spectrum is assessed by:
  (16) $$\begin{array}{c} \rho_{ES_{R}\left(f\right),ES_{A}\left(f\right)}=\frac{\langle ES_{R}\left(f\right),ES_{A}\left(f\right)\rangle }{\sqrt{\langle ES_{R}\left(f\right),ES_{R}\left(f\right)\rangle }\sqrt{\langle ES_{A}\left(f\right),ES_{A}\left(f\right)\rangle }}\\ \;=\frac{\overset{N}{\underset{i=1}{\text{∑}}}ES_{R}\left(f_{i}\right)\times ES_{A}\left(f_{i}\right)}{\sqrt{\overset{N}{\underset{i=1}{\text{∑}}}ES_{R}\left(f_{i}\right)\times ES_{R}\left(f_{i}\right)}\sqrt{\overset{N}{\underset{i=1}{\text{∑}}}ES_{A}\left(f_{i}\right)\times ES_{A}\left(f_{i}\right)}} \end{array}$$

### 3.3. Parameter Identification and Locomotive Bearing Fault Detection

The number and the diameter of the rolling elements in the locomotive bearings are represented by *Z* and *d*, respectively. *D_m_* is the pitch diameter, *α* denotes the contact angle of the bearing, and *f_n_* denotes the rotational frequency. The ball pass frequency of the outer race (BPFO) can be obtained by:

(17) $$\text{BPFO}=\frac{1}{2}(1-\frac{d}{D_{m}}\cos\alpha )f_{n}\;Z$$

If the surface of the outer race suffers a defect, then every time the rolling element passes through the crack, periodic impulses will be created with interval *Δt* as:

(18) $$\Delta t=\frac{1}{\text{BPFO}}$$

Similarly, the ball pass frequency in the inner race (BPFI) is given by:

(19) $$\text{BPFI}=\frac{1}{2}(1+\frac{d}{D_{m}}\cos\alpha )f_{n}\;Z$$

Therefore, the inner race fault characteristic frequency is equivalent to *BPFI*. The optimal parameters to obtain the local maximal envelope spectrum correlation coefficient are then identified, as discussed in Section 3.2. The identified impact period in the Doppler transient model is the related bearing fault impact interval. The fault type can be determined by referring to the calculated theoretical fault-related impact intervals.

## 4. Simulation Validation of the Proposed Method

A simulated signal contaminated by noise is investigated to confirm the effectiveness of the proposed Doppler transient model in recognizing the impact intervals embedded in fault signals. The simulated signal is represented as:

(20) $$x\left(t\right)=e^{-300\pi \mathrm{mod}(t,2/125)}\cos(2000\pi t)+n(t),t=0:1/50000:12400/50000$$

**Figure 4.**
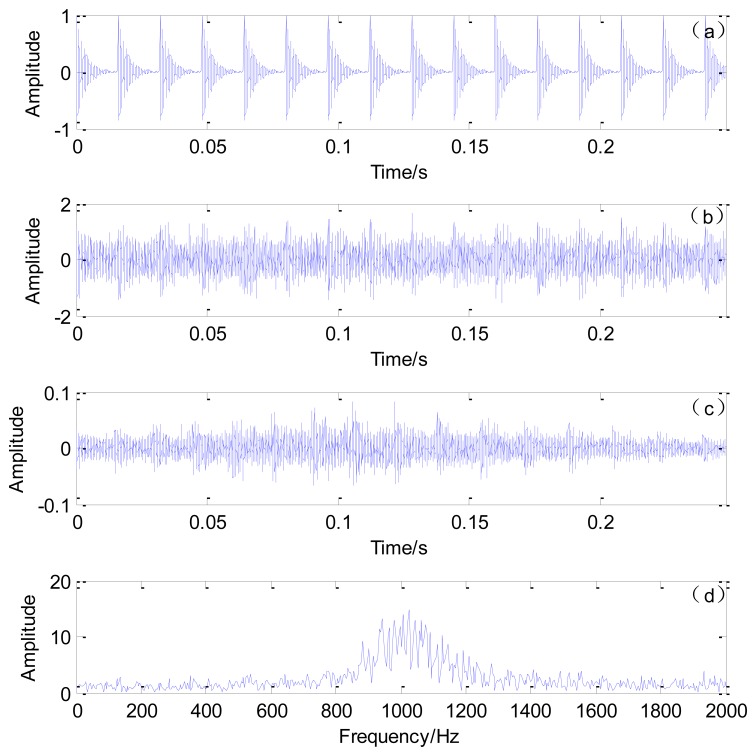
Simulated signals. (**a**) Simulated signal with periodical impacts; (**b**) polluted signal; (**c**) Doppler-distorted signal and (**d**) the Fourier spectrum of the Doppler-distorted signal.

The sampling frequency is 50,000 Hz and the impact interval embedded in the simulated signal is 0.016 s. The number of data points is 12,401. A randomly distributed noise *n(t)* is added to the simulated signal. The simulated and polluted signals are illustrated in Figure 4a,b, respectively.

**Figure 5.**
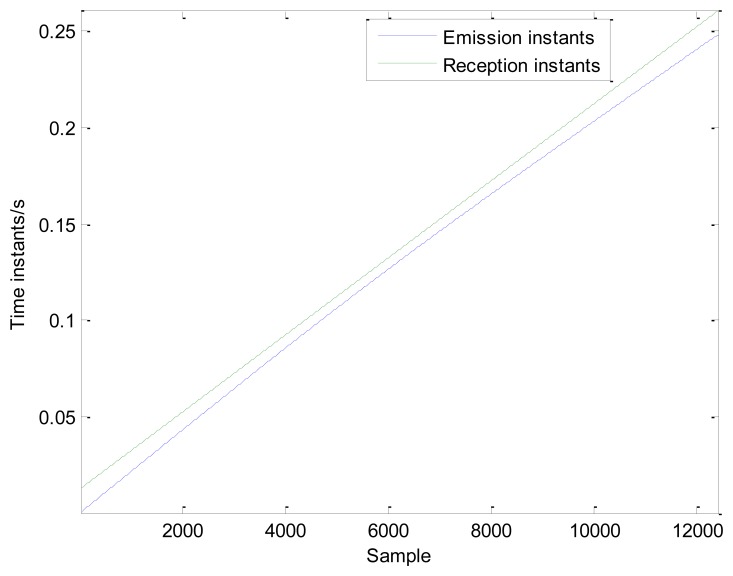
The emission and reception time instants.

To simulate the actual Doppler distortion caused by the relative movement between the moving sound source and the receiver, Doppler distortion is added to the simulated signal according to the procedures specified in Section 3.1. The parameters in Figure 3 are established as follows: *S* = 8 m, *r* = 2 m, *V_s_* = 30 m/s, and *V_sw_* = 340 m/s. The reception time instants are calculated. The initial moment *t_0_* is the propagation time for the sound wave from the initial position to the microphone and *t*_0_= 0.0124 s. Thus the reception time instants {*t_R_*} = {0.0124:1/50,000:0.0124 + 12,400/50,000). The emission time instants are calculated by Equation (7). The emission and reception time instants are shown in Figure 5. The interpolation and amplitude modulation are conducted according to steps 2 and 3 described in Section 3.1. The distorted signal is shown in Figure 4c. Both the impact moments and their amplitudes have changed due to the Doppler effect. These phenomena will significantly affect the recognition of fault-related impacts, thereby enhancing difficulties during fault diagnosis.

**Figure 6.**
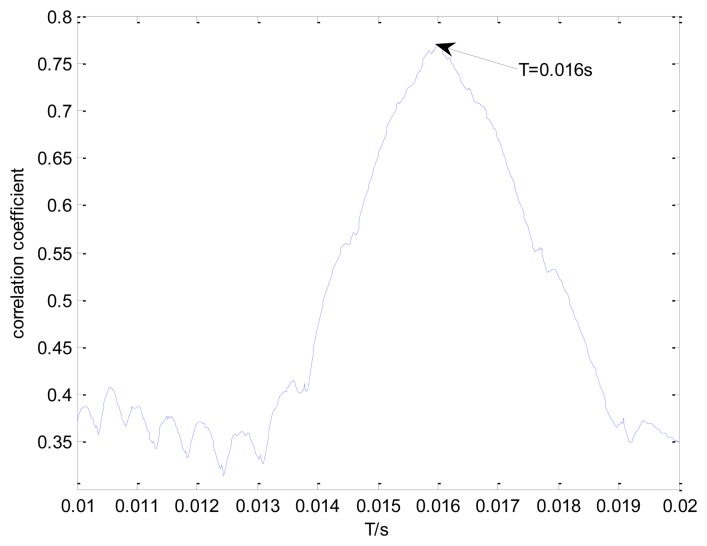
Maximal correlation coefficients for different elements from the set *T*.

The proposed detection method is applied to the Doppler-distorted signal. The transient model is first constructed according to Equation (3). Its parameters require optimization from the sets *T*, *F*, and *Z*. The selection of these sets is crucial, as a larger interval range and a smaller parameter subset step will give a more accurate result. However, this will also result in excessive computational time and decrease the efficiency of the method. Hence, a balance between efficiency and accuracy should be guaranteed. The parameter subsets of *F* and *T* are uniform, as shown in Equations (1) and (2). The range of *F* is set at {800:10:1200}, which is drawn from the Fourier spectrum of the distorted signal. The subset of *Z* is non-uniform to provide higher resolution at lower damping ratio values, so that the efficiency of the method can be retained. Hence, the range of subset Z is selected as {{0.005:0.001: 0.03}{0.04:0.01:0.1}{0.2:0.1:0.9}} which have small steps in the low value range and large steps in the high value range. The impact interval of the transient model is searched from the set *T*, which is selected as {500/50,000:1/50,000:1,000/50,000}. The grid of the model parameters is constructed according to *F* and *Z* for each element from set *T*. When a group of parameters is determined, the transient model Doppler distortion is performed according to the procedures discussed in Section 3.1 to obtain the Doppler transient model. The envelope spectrum correlation between the Doppler transient model and the simulated Doppler distorted signal is assessed. Figure 6 shows the maximal correlation coefficients for the different elements from set *T*.

When the impact interval of the Doppler transient model is determined to be 800/50,000 = 0.016 s, the maximal correlation coefficient of the envelope spectrum between the Doppler transient model and the simulated Doppler distorted signal can be obtained. The optimal parameters *f*=900 and ζ =0.05 when the element 800/50,000 = 0.016 s is determined from set *T* are thus considered the best parameters for the Doppler transient model.

**Figure 7.**
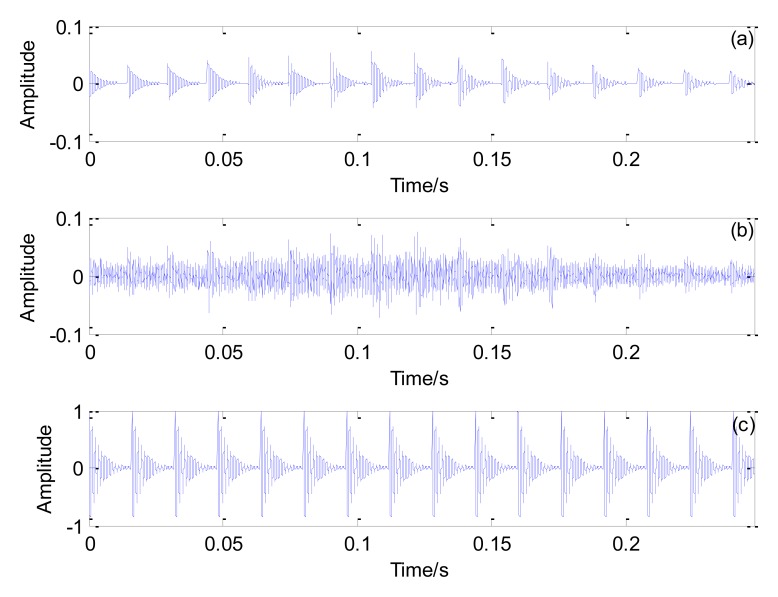
Results obtained by the proposed method: (**a**) the optimal Doppler transient model; (**b**) the simulated Doppler distorted signal; (**c**) the optimal periodical transient model.

The optimal Doppler transient model and the simulated Doppler distorted signal are shown in Figure 7. Thus, after parameter optimization, the optimal Doppler transient model's impact interval matches that of the simulated distorted signal. The impact interval of the original transient model is 800/50,000 = 0.016 s, as shown in Figure 7c. Therefore, the impact interval of the simulated Doppler distorted signal is determined successfully.

## 5. Application of the Proposed Method to Real Locomotive Bearing Fault Diagnosis

**Figure 8.**
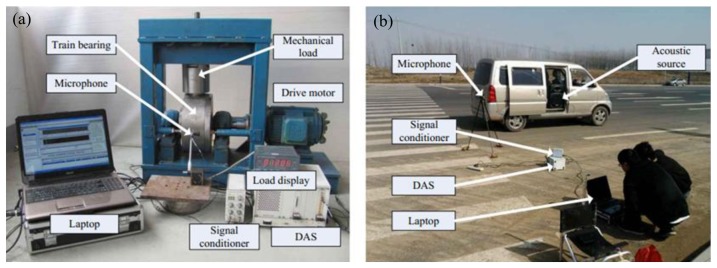
Experimental setup for acoustic signal acquisition with the Doppler effect: (**a**) test bench of the first experiment and (**b**) actual scene of the second experiment.

Real locomotive bearing fault signals suffering from the Doppler effect are analyzed to further validate the performance and applicability of the proposed method. Two sequential experiments are conducted indoors and outdoors to obtain a Doppler-distorted acoustic signal. In the first experiment, the acoustic signals of locomotive bearings with an inner race defect and an outer race defect are acquired through the microphone. The collected acoustic signals are embedded with the Doppler effect in the second experiment. The test rigs for these experiments are illustrated in Figure 8.

As shown in Figure 8a, the test rig is composed of a drive motor, two supporting pillow blocks (mounted with a healthy bearing), and a bearing [NJ(P)3226XI] for testing, which is loaded on the outer race through a worm-and-nut and an adjustable loading system installed in the radial direction. A 4944-A-type microphone from the B&K Company (Copenhagen, Denmark) is mounted adjacent to the outer race of the defective bearing to measure its acoustic signals. An advanced data acquisition system (DAS) by National Instruments (Austin, TX, USA) is used to perform data acquisition. The parameters of the test bearings are listed in Table 1. Some parameters used in the experiment are listed in Table 2.

**Figure 9.**
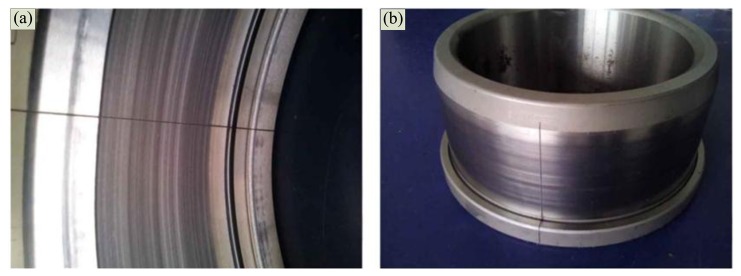
Artificial defects on the (**a**) outer race and (**b**) on the inner race of the bearing.

Figure 8b shows a realistic setup of the second experiment, which is represented by the model illustrated in Figure 3. The parameters are established as follows: *S* = 8 m, *r* = 2 m, *V_s_* = 30 m/s, and *V_sw_* = 340 m/s. The acoustic source is mounted in a moving vehicle, and the microphone and DAS from the first experiment were used. To simulate the locomotive bearing fault, an artificial crack with a width of 0.18 mm is made with a wire-electrode cutting machine on the surfaces of either the outer race and inner race, as shown in Figure 9. The Doppler-distorted inner race fault and outer race fault signals are obtained in these experiments. The proposed method is then used to detect the fault-related impact intervals.

**Figure 10.**
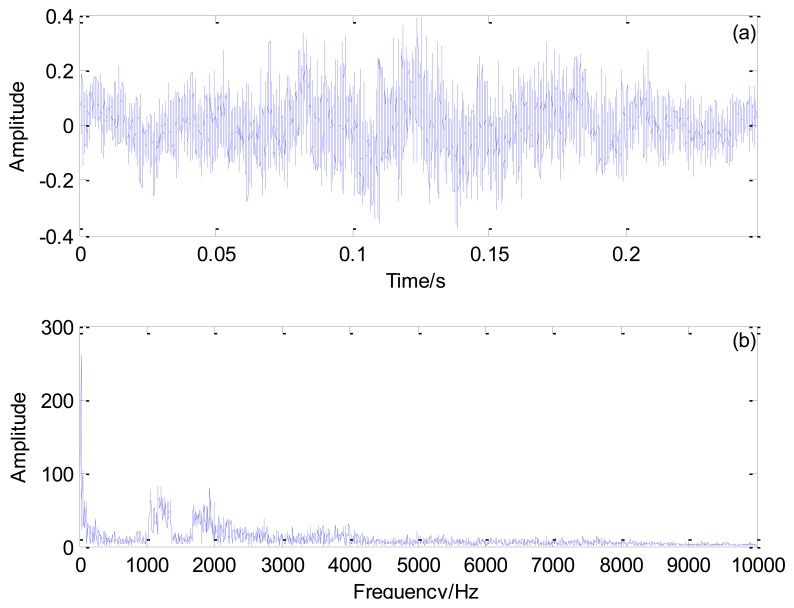
Outer race fault signal of the locomotive bearing under the loading of 3 t: (**a**) time domain signal and (**b**) its spectrum.

Figure 10 shows the Doppler-distorted outer race fault signal under the loading of 3 t and its spectrum. As computed by Equation (17), the outer race characteristic frequency is 138.74 Hz and the periodical impact interval is 0.0072 s.

To detect the characteristic interval embedded in the fault signal, a periodical transient model with parameters adjustable using Equation (3) is constructed. Doppler distortion is added to the transient model according to the procedures described in Section 3.1. The correlation of the envelope spectrum between the Doppler transient model and the outer race fault signal for a selected group of parameters is assessed. The constructed Doppler transient models demonstrate different correlation coefficients with the real locomotive bearing fault signal in the frequency domain for different pairs of parameters.

**Figure 11.**
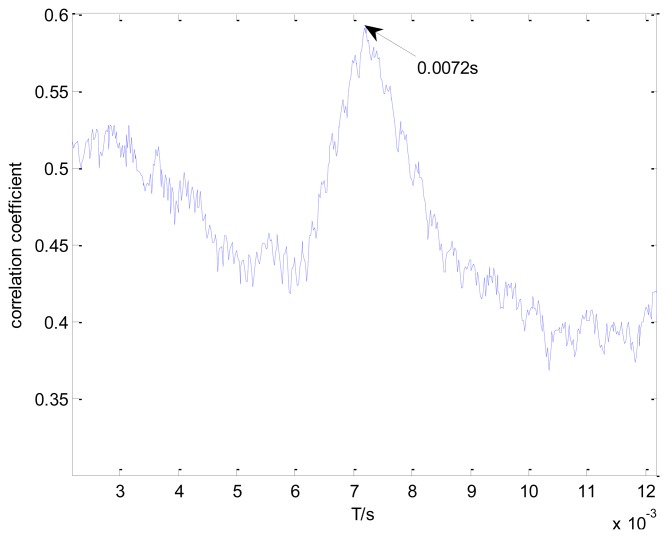
Maximal correlation coefficients for different elements from set *T*.

**Figure 12.**
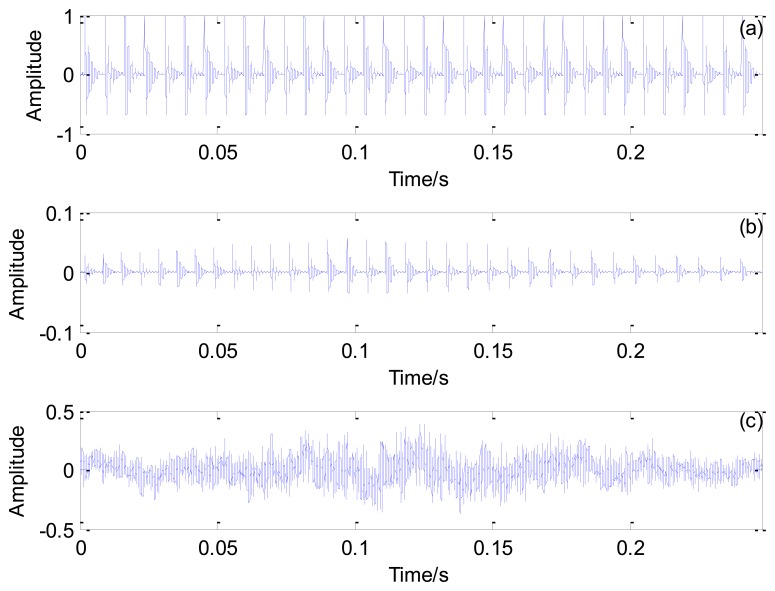
Results obtained using the proposed method: (**a**) the optimal periodical transient model; (**b**) the optimal Doppler transient model and (**c**) the outer race fault signal.

Figure 11 shows the maximal correlation coefficients for each selected impact period. The maximal correlation coefficient reaches its global maximum when the impact period is 0.0072 s, which is the real bearing fault-related impact interval. The optimal transient model and its Doppler-distorted model are shown in Figure 12a,b, respectively. A comparison between the optimal Doppler transient model and the real locomotive bearing fault signal in Figure 12c indicates that the proposed transient model correctly reveals the embedded fault-related impact intervals.

The conventional method, which conducts the correlation assessment in the time domain, is applied to the problem for comparison. A transient model with parameters requiring optimization from sets *T*, *F*, and *Z* is constructed. The correlation between the transient model and the Doppler distorted signal is assessed in the time domain [19] instead of the frequency domain.

**Figure 13.**
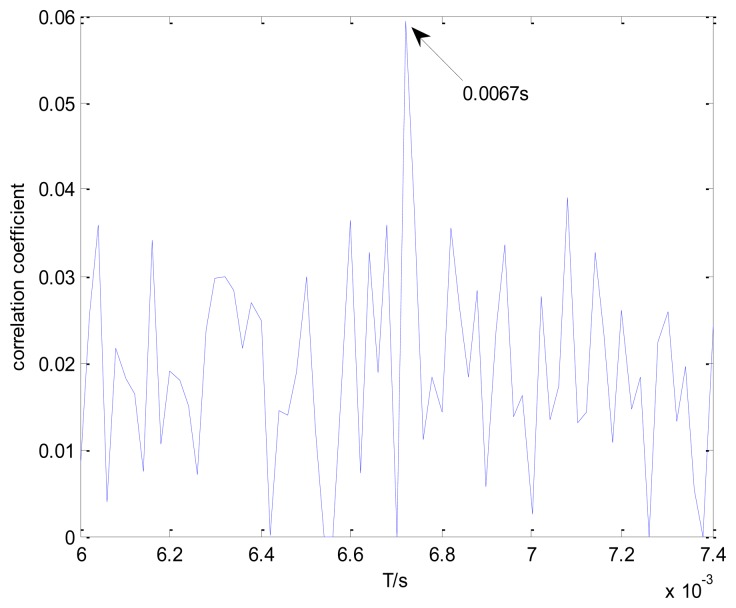
Maximal correlation coefficients for different elements from set *T* using the conventional method.

Figure 13 shows the maximal correlation coefficients for the different elements from set *T*. The maximal correlation coefficient is obtained when the impact period is 0.0067 s. However, this is not the real outer race fault-related impact interval. The values of the correlation coefficients are much smaller than those in Figure 11. Hence, the conventional method is not applicable to this problem.

**Figure 14.**
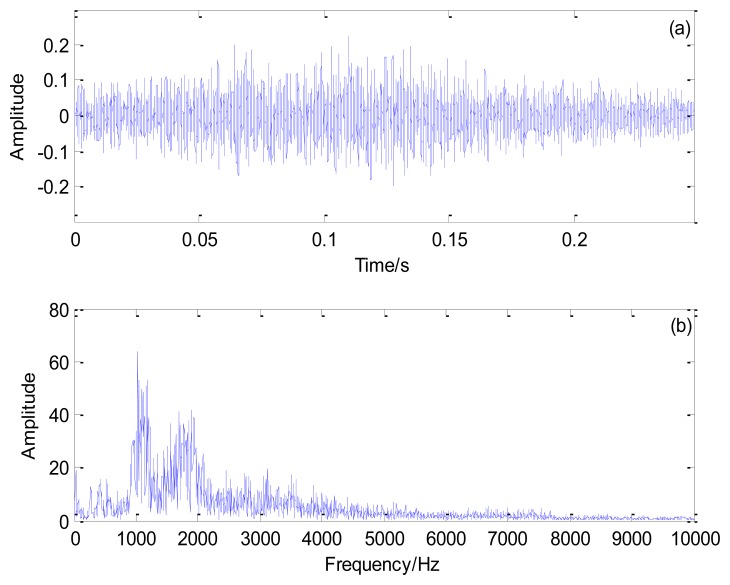
Outer race fault signal of the locomotive bearing under the loading of 1 t: (**a**) time domain signal and (**b**) its spectrum.

**Figure 15.**
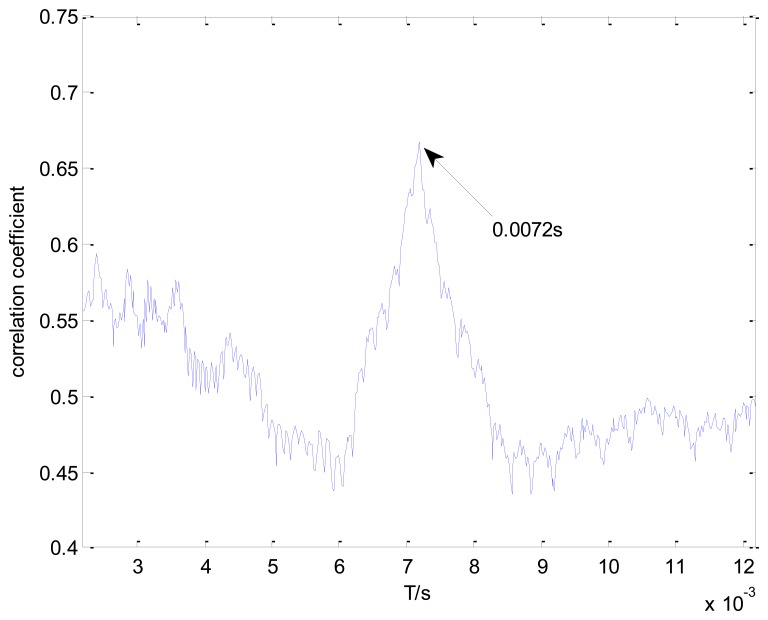
Maximal correlation coefficients for different elements from set *T*.

**Figure 16.**
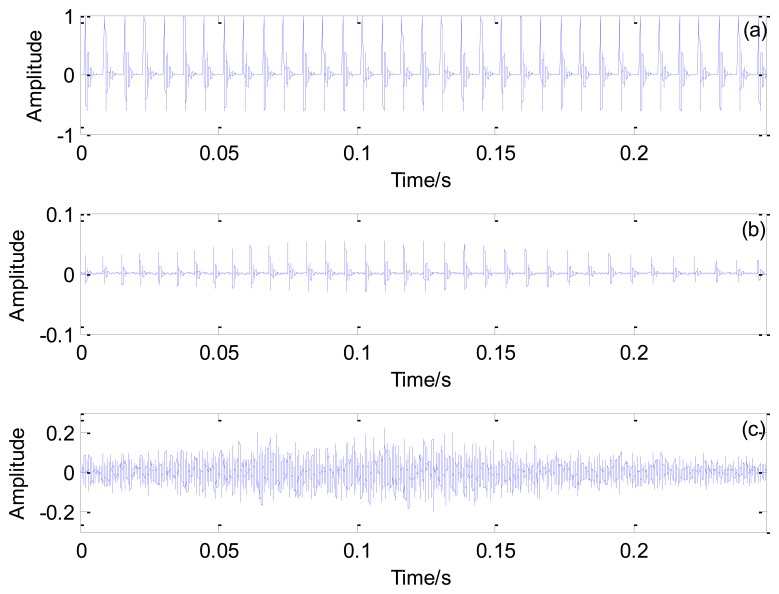
Results for a loading of 1*t* obtained using the proposed method: (**a**) the optimal periodical transient model; (**b**) the optimal Doppler transient model and (**c**) the outer race fault signal.

An outer race fault signal under a different loading, 1 t, is analyzed. Figure 14 shows the Doppler-distorted outer race fault signal under the loading of 1 t and its spectrum. This signal is processed according to the procedures in Figure 2, the results of which are shown in Figures 15 and 16. The proposed method correctly reveals the fault-related impact intervals under this different working condition.

**Figure 17.**
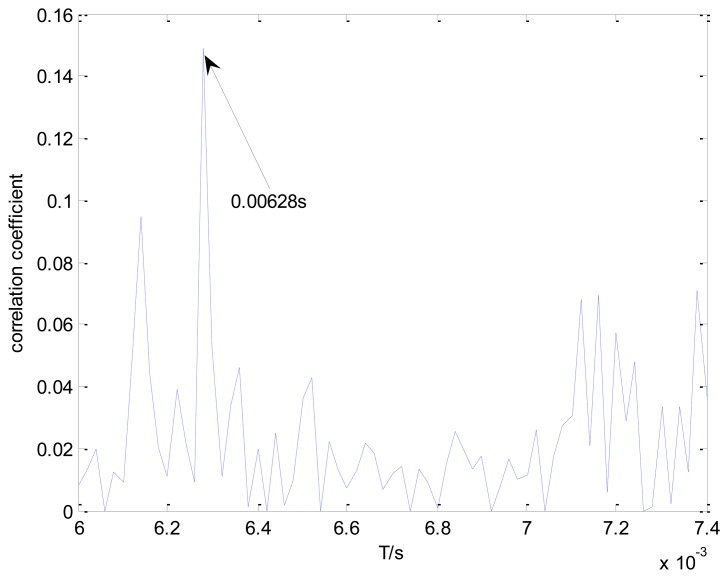
Maximal correlation coefficients for different elements from set *T*, using the conventional method and a loading of 1 t.

The conventional method in the time domain is again used for a comparative analysis. Figure 17 shows the maximal correlation coefficients between the transient model and the real bearing fault signal for the different elements from set *T*. The conventional method fails to identify the locomotive bearing fault-related impact interval, as the optimal impact interval found is 0.00628 s instead of 0.0072 s.

**Figure 18.**
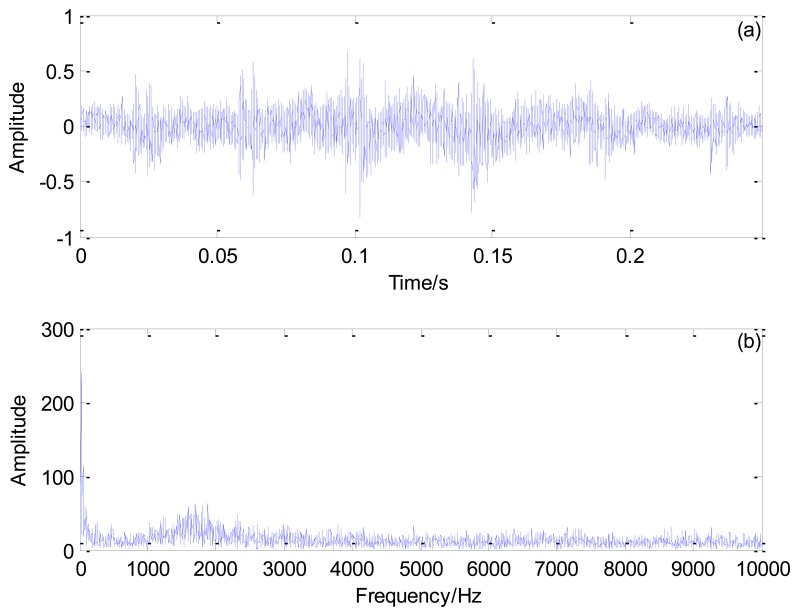
Inner race fault signal of the locomotive bearing: (**a**) time domain signal and (**b**) its spectrum.

The actual inner race fault signal shown in Figure 18 is analyzed using the proposed method. Using Equation (19), the inner race fault characteristic impact interval is calculated as 0.0051 s.

**Figure 19.**
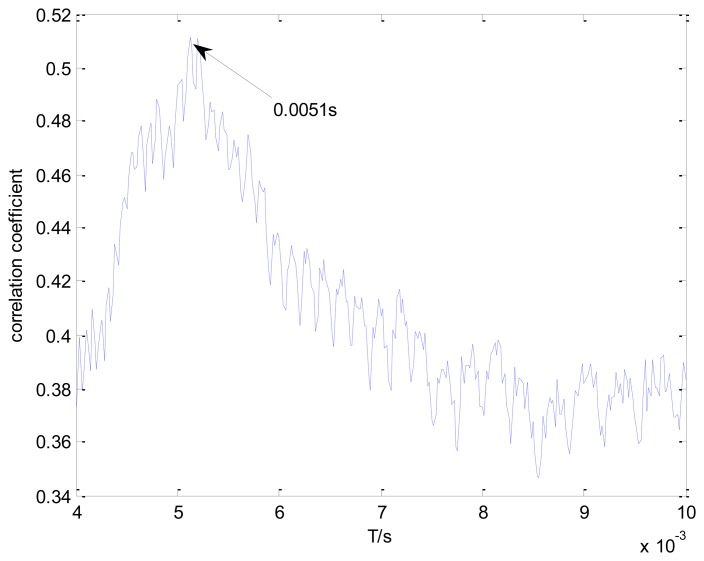
Maximal correlation coefficients for different elements from set *T*, using the proposed method on the inner race fault signal.

A transient model with optional parameters is established to recognize the locomotive bearing fault. The Doppler distortion is added into the constructed model. The maximal correlation coefficients for every selected impact period after parameter optimization are shown in Figure 19. The global maximal correlation coefficient is obtained when the impact period for the established transient model is set as 0.0051 s.

**Figure 20.**
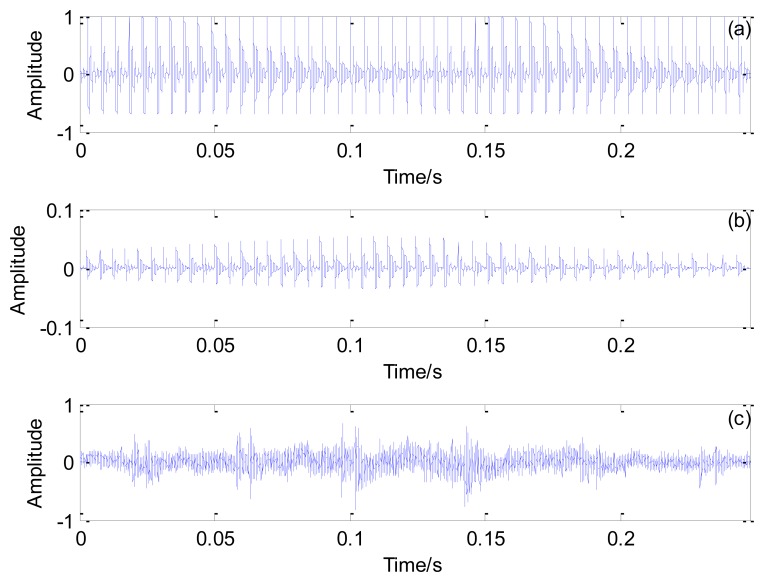
Results obtained by using the proposed method: (**a**) the optimal periodical transient model; (**b**) the optimal Doppler transient model and (**c**) the inner race fault signal.

Figure 20a–c illustrates the optimal transient model, the Doppler distorted transient model, and the real inner race fault signal, respectively. Based on the results in Figure 20, the inner race fault-related impact interval embedded in the actual fault signal is successfully revealed by applying the proposed method.

**Figure 21.**
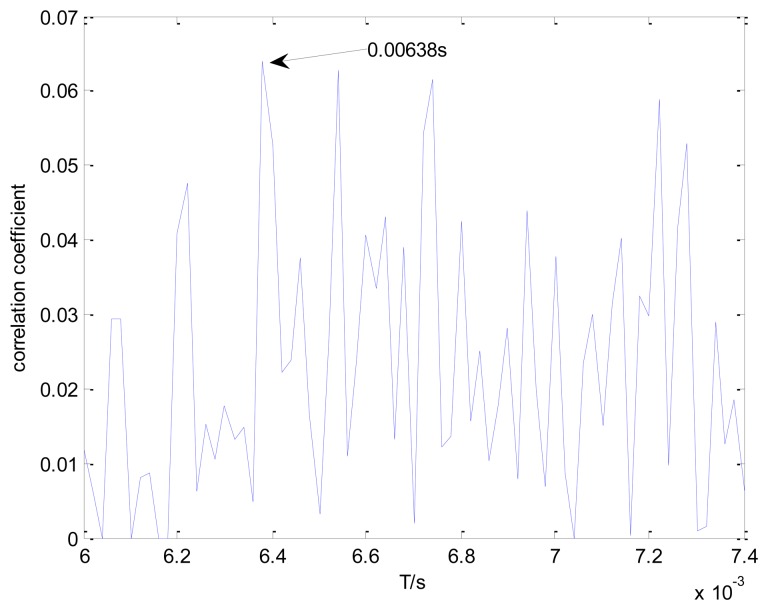
Maximal correlation coefficients for different elements from set *T*, using the conventional method on the inner race fault signal.

A comparative analysis between the proposed method and the conventional method is also conducted on the inner race fault signal processing. Figure 21 presents the maximal correlation coefficients between the transient model and the real locomotive bearing fault signal in the time domain. The inner race fault-related impact interval is not successfully recognized, as the conventional method incorrectly selects the impact period *T* = 0.00638 s. The performance and superiority of the proposed method is therefore validated by these specific case studies and comparative analyses.

## 6. Conclusions

In this study, a new Doppler transient model based on the Laplace wavelet and a spectrum correlation assessment is proposed for diagnosing locomotive bearing faults. The proposed scheme includes Laplace wavelet transient model construction, Doppler distortion, spectrum correlation assessment, and parameter optimization. After implementing the proposed method, the fault-related impact interval can be successfully determined using on the optimal Doppler transient model.

The Laplace wavelet is used as the impact base function due to its superior ability to match actual bearing fault impulses. A periodical transient model based on the Laplace wavelet is constructed. The parameters of the model require optimization to properly match the real locomotive bearing fault impact interval.

Through acoustical theoretical analysis, a procedure for adding the Doppler effect to the constructed periodical transient model is proposed to simulate the Doppler distortion experienced by real locomotive bearing fault signals.

A new criterion is established to choose proper parameters during Doppler transient model construction. Correlation analysis is conducted between the envelope spectrum of the established Doppler transient model and the locomotive bearing fault signal. The parameters for obtaining the maximal correlation coefficient are found to be the optimal parameters for the model. Hence, the impact interval in the optimal Doppler transient model is recognized as the fault-related impact interval.

The results obtained by investigating both simulated signals and locomotive bearing fault signals indicate that the proposed method exhibits satisfactory performance in analyzing Doppler-distorted locomotive bearing acoustical fault signals. The proposed method could be developed further for use in a wayside train condition monitoring system.

## Figures and Tables

**Table 1.** Specifications of the testing bearing.

**Type**	**NJ(P)3226XI**
Diameter of the outer race	250 mm
Diameter of the inner race	130 mm
Pitch diameter (*D*)	190 mm
Diameter of the roller (*d*)	32 mm
Number of the roller (*z*)	14 mm

**Table 2.** Parameters used in the first experiment.

**Loading**	3 t, 1 t
**Rotating Speed**	1,430 rpm
**Sampling Frequency**	50 kHz
